# Assessing the Utility of a Knowledge Based Algorithm for Classifying Alzheimer disease and Mild Cognitive Impairment in the Recruitment and Retention for Alzheimer’s Disease Diversity Cohorts in the ADSP (READD‐ADSP) Project

**DOI:** 10.1002/alz.091433

**Published:** 2025-01-03

**Authors:** Andrew F. Zaman, Pedro R. Mena, Katalina F McInerney, Katrina Celis, Larry D. Adams, Tina Duke, Jose Javier Sanchez, Jenny Chavez, Jennifer Cespedes, Takiyah D. Starks, Renee A. Laux, Kara L. Hamilton‐Nelson, Farid Rajabli, Goldie S. Byrd, Christiane Reitz, Giuseppe Tosto, Paula K. Ogrocki, Allison M Caban‐Holt, Jonathan L. Haines, Jeffery M. Vance, Margaret A Pericak‐Vance, Michael L. Cuccaro

**Affiliations:** ^1^ John P. Hussman Institute for Human Genomics, University of Miami Miller School of Medicine, Miami, FL USA; ^2^ University of Miami Miller School of Medicine, Miami, FL USA; ^3^ Gertrude H. Sergievsky Center, Taub Institute for Research on the Aging Brain, Departments of Neurology, Psychiatry, and Epidemiology, College of Physicians and Surgeons, Columbia University, New York, NY USA; ^4^ Maya Angelou Center for Health Equity, Wake Forest University School of Medicine, Winston‐Salem, NC USA; ^5^ Department of Population and Quantitative Health Sciences, Case Western Reserve University, Cleveland, OH USA; ^6^ Department of Neurology, Case Western Reserve University School of Medicine, Cleveland, OH USA; ^7^ Department of Neurology, University Hospitals Cleveland Medical Center, Cleveland, OH USA; ^8^ Cleveland Institute for Computational Biology, Case Western Reserve University, Cleveland, OH USA; ^9^ Dr. John T. Macdonald Foundation Department of Human Genetics, University of Miami Miller School of Medicine, Miami, FL USA; ^10^ 1501 NW 10th Avenue, Miami, FL USA

## Abstract

**Background:**

Over 13,000 individuals from both domestic and African sites will be collected for the READD‐ADSP study. Adjudicating this number of individuals is challenging, so we evaluated knowledge‐based decision tree algorithms to predict clinical diagnoses using nationally representative norms and standard cut‐offs. Additional models were constructed using culturally adjusted cut‐offs, domain average cut‐offs, and exclusion of the Trail Making Test (TMT) which performed poorly. Our aim was to evaluate the accuracy of these decision tree models in the READD‐ADSP dataset.

**Method:**

Clinically adjudicated diagnoses by at least two neuropsychologists and/or neurologists were available for 150 individuals from the READD‐ADSP sample (44%‐Unaffected, 36%‐MCI, 9.3%‐AD, 2.7%‐Dementia not AD, 8%‐Cognitively Impaired– Vascular; 19% Hispanic, 81% African‐American, 76%‐Female, Age: 73.0 years, Education: 13.7 years). Eight models were used to classify our sample into the diagnostic categories above and varied in test cut‐offs (standard vs. culturally adjusted), use of domain average cut‐offs (no average vs. average), and use of TMT (inclusion vs. exclusion). Model accuracy was assessed using the F1 score (which balances precision and sensitivity). F1 scores >0.9 are considered excellent, >0.8 are good, and those between 0.6‐0.8 are acceptable.

**Result:**

The two models that used culturally adjusted cut‐offs and domain average cut‐offs performed best (with TMT, F1 = 0.81; without TMT, F1 = 0.78). All other models had F1 scores ranging from 0.63‐0.73. While the two best models were similarly accurate based on F1 scores, the difference in the frequency of diagnoses between them was notable. In the model that excluded TMT, 38% of the sample were classified as Unaffected vs. 30% in the model that included TMT; (41% and 47% were classified MCI, respectively).

**Conclusion:**

Our findings show that culturally sensitive adjusted cut‐offs and inclusion of domain average cut‐offs improved the accuracy of decision tree model classifications. These findings suggest that implementation of decision tree model approaches in the READD‐ADSP dataset can accurately and efficiently classify individuals. Finally, we plan improve overall accuracy by using a hybrid method where models classify individuals who are clearly Unaffected or AD while more complex cases will be assigned to human adjudication.

